# The opposing roles of Wnt-5a in cancer

**DOI:** 10.1038/sj.bjc.6605174

**Published:** 2009-07-14

**Authors:** S L McDonald, A Silver

**Affiliations:** 1 Colorectal Cancer Genetics Group, Institute for Cell and Molecular Sciences, Barts and The London School of Medicine and Dentistry, Queen Mary University, 4 Newark Street, Whitechapel, London E1 2AT, UK

**Keywords:** Wnt signalling, Wnt-5a

## Abstract

Wnt-5a is one of the most highly investigated non-canonical Wnts and has been implicated in almost all aspects of non-canonical Wnt signalling. In terms of cancer development, Wnt-5a has, until recently, lived in the shadow of its better-characterised relatives. This was largely because of its apparent inability to transform cells or signal through the canonical *β*-catenin pathway that is so important in cancer, particularly colorectal cancer. Recent work in a wide range of human tumours has pointed to a critical role for Wnt-5a in malignant progression, but there is conflicting evidence whether Wnt-5a has a tumour-promoting or -suppressing role. Emerging evidence suggests that the functions of Wnt-5a can be drastically altered depending on the availability of key receptors. Hence, the presence or absence of these receptors may go some way to explain the conflicting role of Wnt-5a in different cancers. This review summarises our current understanding of Wnt-5a and cancer.

Wnt-5a is 1 of 19 Wnt proteins that make up the family of secreted lipid-modified glycoproteins that show a highly regulated pattern of expression and has distinct roles during development and tissue homoeostasis. The importance of Wnt-5a in multiple developmental pathways is illustrated by the phenotype of *Wnt-5a*^*−/−*^ mice, which die at birth and show many defective features such as truncated bodies, facial abnormalities and short deformed limbs (Oishi *et al*, 2003). Wnts have been implicated in oncogenesis as early studies showed that Wnt-1 was overexpressed in mammary epithelial adenocarcinomas as a result of integration of the mouse mammary tumour virus. Since then, deregulated expression of Wnts and Wnt signalling has been linked to multiple forms of epithelial cancer (Giles *et al*, 2003). However, on account of the inability of Wnt-5a to transform cells, its role in cancer promotion was not immediately apparent. Nevertheless, recent work has pointed to a critical role of Wnt-5a in malignant progression (Jonsson *et al*, 2002; Huang *et al*, 2005; Roman-Gomez *et al*, 2007; Da Forno *et al*, 2008), but whether it is afforded by a tumour-suppressing effect or an oncogenic effect is questionable. The fact that Wnt-5a has been described as both challenges the simplistic classification of genes as tumour promoters or suppressors. In this review, we discuss the opposing roles of Wnt-5a in cancer development and show that an account of the Wnt-5a cellular and signalling context should be taken before a functional classification can be made.

## Wnt signalling pathways

The Wnt signalling pathways are usually activated through binding of the secreted Wnt molecules to the conserved C-terminal cytosolic domain of a family of nine large multipass transmembrane receptors called the Frizzled receptor proteins (Fz). However, Wnts do not signal exclusively through these receptors as others, such as the ROR2 receptor, have been identified (Oishi *et al*, 2003). The interaction between individual Wnts and their specific receptors is thought to dictate the type of downstream signalling pathways that are activated. Accordingly, the Wnts have historically been divided into two classes: those that signal through the ‘canonical’ or the ‘non-canonical’ signalling pathway.

The canonical signalling pathway performs a variety of different functions throughout development. Canonical Wnts are thought to activate a signal-transduction pathway that induces the nuclear accumulation and transcriptional activation of *β*-catenin (Giles *et al*, 2003). A process that can cause duplication of the embryonic axis in Xenopus (x) (xWnt-1, xWnt-3a, xWnt-8a and xWnt-8b) (Du *et al*, 1995) and transform mouse (m) mammary epithelial cells (mWnt-1, mWnt-2, mWnt-3, mWnt-3a) (Shimizu *et al*, 1997), this pathway is essential for the development of dorsal polarity during grastulation (Brannon *et al*, 1997). In addition to a role in normal development, deregulation has been shown to be integral to cancer development and progression by, among other functions, promoting cancer cell proliferation and migration (Giles *et al*, 2003).

The non-canonical signalling pathway is essentially an umbrella term for all Wnt-activated cellular signalling pathways that do not promote *β*-catenin-mediated transcription, and numerous pathways have been identified. In contrast to the canonical Wnts, the non-canonical Wnts do not signal through *β*-catenin, do not cause duplication of the embryonic axis in Xenopus (xWnt-4, xWnt-5A and xWnt-11) (Du *et al*, 1995) and are unable to transform mouse mammary epithelial cells (mWnt-4, mWnt-5a, mWnt-5b, mWnt-6 and mWnt-7b) (Shimizu *et al*, 1997). Furthermore, it is well established that non-canonical Wnts can antagonise the functions of canonical Wnts (Torres *et al*, 1996). A recent study provided further evidence of the opposing roles of different Wnts as *β*-catenin gene targets upregulated in B16 murine melanoma cells treated with Wnt-3a were universally downregulated in B16 cells treated with Wnt-5a (Chien *et al*, 2009). Although involved in many processes (Veeman *et al*, 2003; Semenov *et al*, 2007), non-canonical Wnts are generally considered to control morphogenic movements. The most well-recognised categories of non-canonical signalling are the planar cell polarity pathway (PCP) and the Wnt calcium signalling pathway. Wnt-5a is one of the most highly investigated non-canonical Wnts, and has been shown to be involved in almost all aspects of non-canonical Wnt signalling.

Interestingly, the convention of two independent Wnt pathways has remained for some time, but emerging evidence suggests that the pathways are not as autonomous as originally thought. For instance, although Wnt-5a is thought to primarily function though the non-canonical pathway, it can, under certain circumstances, signal through the canonical pathway. The possibility of interaction between these two pathways may explain in part the uncertainties of the role of Wnt-5a in cancer.

## The role of Wnt-5a in Wnt signalling and implications for cancer

**Figure 1:**
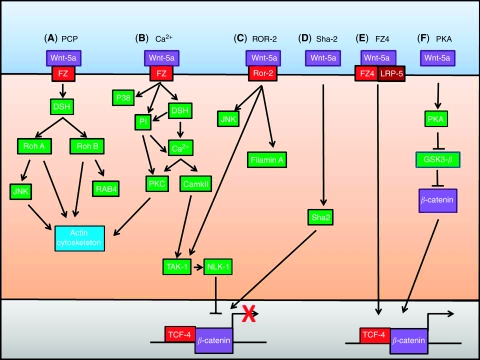
An overview of Wnt-5a signalling. (**A**) Wnt-5a can activate PCP through a process dependent on Roh A and possibly Roh B leading to the control of cellular movement. (**B**) Wnt-5a uses numerous signalling molecules leading to the release of Ca^2+^ resulting in various cellular effects including cell movement and inhibition of the canonical Wnt signalling pathway. (**C**) Wnt-5a can bind the ROR-2 receptor activating JNK and the cytoskeleton as well as inhibiting *β*-catenin/TCF dependent transcription. (**D**) Wnt-5a can inhibit *β*-catenin/TCF-dependent transcription through Shia-1. (**E**) In the presence of FZ4 and LRP-5, Wnt-5a can activate *β*-catenin/TCF-dependent transcription. (**F**) Wnt-5a can activate PKA, which in turn can inhibit GSK-*β* to promote *β*-catenin/TCF-dependent transcription. Figure adapted from Semenov *et al* (2007).

The non-canonical pathway, PCP, was identified in *Drosophila*, and is essential for organising the orientation of cells in tissues. In *Drosophila*, PCP is a complex process involving the spatial organisation of multiple signalling molecules (Jones and Chen, 2007). Signalling results in the activation of a number of cytoskeleton regulators including DAAM1, Rac, Rho and Rho kinase (Jones and Chen, 2007). Although this pathway was identified in *Drosophila*, it is by no means confined to this organism and has also been identified in vertebrates (Green and Davidson, 2007), but the extent of the similarities are unknown (Mlodzik, 2002). Furthermore, vertebrates use PCP to allow cells to undergo convergence and extension movements during organogenesis (Wallingford *et al*, 2002). As a result, PCP is now thought to be essential for the organisation, orientation and morphogenic movements of multiple invertebrate and vertebrate epithelial and mesenchymal cells throughout normal development and is thought to be activated in cancer (Jones and Chen, 2007). Wnt-5a was recently shown to be essential for controlling PCP in vertebrates (Qian *et al*, 2007) (Figure 1A). In addition, Wnt-5a, in the presence of a CXCL12 chemokine gradient, was able to polarise the cellular cytoskeleton of WM239a melanoma cells through a process dependent on dishevelled (DSH), RhoB and Rab4 to promote cellular migration towards the source of the chemokine (Witze *et al*, 2008). Although these reports shed some light on the role of Wnt-5a in PCP, the downstream signalling pathways activated to bring about its effects are still enigmatic, but it is clear that it operates through the cytoskeleton to control cell orientation and movement. This ability of Wnt-5a to promote cell movement has crucial implications for cancer progression.

The second main Wnt-5a-dependent pathway is the calcium-dependent signalling pathway. Here, the non-canonical Wnts can trigger intracellular calcium flux, which can lead to the activation of calcium-dependent signalling molecules such as calmodulin-dependent protein Kinase II (CAMKII) and protein kinase C (PKC) (Kuhl *et al*, 2000b). The pathways activated downstream perform many different tasks, and there is a degree of crossover between the calcium-dependent pathway and the PCP pathway. In contrast with the canonical pathway, this pathway can control the fate of ventral cells in Xenopus (Kuhl *et al*, 2000a). Some of the earliest experiments carried out in Xenopus embryos showed that Xenopus Wnt-5A (Xwnt-5A) was able to activate Rat (r) FZ-2, resulting in intracellular Ca^2+^ release (Slusarski *et al*, 1997b). The components of the signalling pathway upstream of the release of Ca^2+^ are still debated, but it is clear that a number of signalling molecules such as DSH (Sheldahl *et al*, 2003) and p38 (Ma and Wang, 2007) are activated as is G-protein-linked phosphatidylinositol signalling (Slusarski *et al*, 1997a). The Ca^2+^ release is thought to lead to activation of CamKII ensuring correct axis formation and the promotion of ventral cell fate (Kuhl *et al*, 2000a). Studies have also shown that XWnt-5A can bind to rFZ-2 to activate PKC (Sheldahl *et al*, 2003) (Figure 1B). CamKII and PKC activation by Wnt-5a is maintained in higher organisms and is essential for invasion of cancer cells (Weeraratna *et al*, 2002; Dissanayake *et al*, 2007). Therefore, it can be concluded that overexpression of Wnt-5a could have an oncogenic effect by stimulating cancer cell invasion.

In addition to Frizzled receptors, Wnt-5a can also bind and activate the ROR2 tyrosine kinase receptor resulting in the activation of the actin-binding protein, filamin A, and the JNK signalling pathway (Oishi *et al*, 2003; Nomachi *et al*, 2008) (Figure 1C). A potential role for these pathways in cancer was established when Wnt-5a was shown to signal through ROR2 to induce cellular migration and invasion in murine fibroblast NIH3T3 cells (Nomachi *et al*, 2008); if the same occurred in cancer cells, oncogenic potential could be conferred by Wnt-5a through the promotion of cancer cell invasion.

In addition to activating non-canonical signalling, Wnt-5a is also able to inhibit the activation of the canonical signalling pathway by a number of mechanisms, either by calcium signalling through CamKII (Torres *et al*, 1996) or through the ROR2 signalling pathways (Mikels and Nusse, 2006) (Figure 1 B and C). In turn, these pathways can stimulate the TAK1–NLK pathway to phosphorylate (Winkel *et al*, 2008) and inactivate the active *β*-catenin transcription complex (Ishitani *et al*, 2003). Another proposed mechanism for the inhibition of *β*-catenin-mediated transcription is through the upregulation of Sha2, which occurs in response to Wnt-5a-mediated calcium release in APC mutant cells (MacLeod *et al*, 2007) (Figure 1D). In accordance with this, Wnt-5a has been shown to reduce the activation of *β*-catenin-mediated transcription in HCT116 (Ying *et al*, 2008) and HT-29 colon cancer cell lines (MacLeod *et al*, 2007). Through inhibiting the activation of canonical Wnt signalling, the expression of Wnt-5a is likely to confer a tumour-suppressive role in tumours that rely on canonical signalling for survival.

Although the ability of Wnt-5a to inhibit the activation of *β*-catenin-mediated transcription is well established, there is evidence to suggest that in the presence of FZ-4 and LRP-5 and the absence of ROR2, Wnt-5a can stimulate *β*-catenin transcriptional activation (Mikels and Nusse, 2006) (Figure 1E). Research has also shown that Wnt-5a can activate phospho kinase A (PKA) in primary cultured human dermal fibroblasts, which in turn can inactivate GSK3-*β* resulting in stabilisation and nuclear accumulation of *β*-catenin, and concomitantly promote the activation of an important cotranscription factor of *β*-catenin, the CRE-binding protein (CREB) (Torii *et al*, 2008) (Figure 1F). Therefore, Wnt-5a, in the presence of specific FZ isoforms, could promote tumour growth by activation of the cancer-promoting canonical Wnt signalling pathway. Nevertheless, it is important to note that this influence on *β*-catenin-mediated transcription may only be effective in some cell types as Wnt-5a was shown to have no effect on the activation of transcription in MCF-7 breast cancer cells (Pukrop *et al*, 2006).

Wnt-5a is one of the most highly investigated non-canonical Wnts, and has been shown to be involved in almost all aspects of the non-canonical Wnt signalling pathway. Wnt-5a has ample opportunity, therefore, to influence cancer development.

## Expression of Wnt-5a in cancer

Unsurprisingly, given its functional promiscuity, investigations to elucidate the role of Wnt-5a in cancer have shown paradoxical results and studies indicate that it may have a tumour suppressing or an oncogenic effect depending on the cancer type (Table 1). A large number of studies have indicated that Wnt-5a commands a tumour-suppressing effect, and it was shown to be downregulated in a number of different cancers such as colorectal cancer (Dejmek *et al*, 2005a; Ying *et al*, 2008), neuroblastoma (Blanc *et al*, 2005), ductal breast cancer (Jonsson *et al*, 2002; Dejmek *et al*, 2005b) and leukaemias (Liang *et al*, 2003; Roman-Gomez *et al*, 2007; Ying *et al*, 2007). Downregulation of Wnt-5a has been associated with higher tumour grade (Kremenevskaja *et al*, 2005; Dejmek *et al*, 2005a; Liu *et al*, 2008) and was shown to be an independent factor indicating poor prognosis in a number of different tumour subtypes (Jonsson *et al*, 2002; Roman-Gomez *et al*, 2007). These results suggest that for cancer to progress, Wnt-5a must be actively silenced, a characteristic feature of tumour suppressors. This tumour-suppressive role was further evidenced by studies that reintroduced Wnt-5a into SW480 colorectal cancer or thyroid cancer FTC-133 cell lines resulting in decreased invasion, migration, colonogenicity and proliferation (Kremenevskaja *et al*, 2005; Dejmek *et al*, 2005a). Further evidence of a potential tumour-suppressive role was shown by a synthetic peptide synthesised to mimic the biological properties of Wnt-5a. This peptide could reduce the invasion of breast cancer cell lines *in vitro* and inhibited the metastatic spread of 4T1 breast cancer cells from the mammary fat pad to the lungs and liver by 70–90% in athymic BALB/c mice (Safholm *et al*, 2008).

Most studies have involved limited sample sets in terms of numbers and a significant number have not detailed expression at both the RNA and protein levels. Studies with much larger sample sets will provide the necessary statistical power to validate the extent of the downregulation of Wnt-5a in cancer. However, the current data do indicate that reduced expression is likely to occur in over half of each of the tumour types investigated. As the majority of these studies investigated Wnt-5a protein expression, a mechanism for this loss of expression has not been established. It is possible, however, that epigenetic regulation is involved, as methylation of the Wnt-5a promoter was identified in a large proportion of lymphoblastic leukaemia patients and in colorectal cancer patients (Roman-Gomez *et al*, 2007; Ying *et al*, 2007, 2008). The precise mechanisms governing cancer promotion caused by Wnt-5a downregulation are still unknown. As Wnt-5a can counteract the effects of canonical Wnt signalling, it seems clear that its downregulation would be advantageous to cancers driven by canonical Wnt signalling. However, the mechanisms that inhibit tumour growth in tumours without active canonical Wnt signalling remain unclear. It has recently been determined that Wnt-5a can promote the association of *β*-catenin and E-cadherin complexes on the cell membrane leading to increased cellular adhesion (Medrek *et al*, 2009). Therefore, reducing Wnt-5a expression may diminish cellular adhesion through reducing membrane-bound E-cadherin. This explanation is especially persuasive as reduced WNT-5a expression has been identified in a large proportion of ductal breast cancers (Jonsson *et al*, 2002; Dejmek *et al*, 2005b), which rarely have inactivation mutations in E-cadherin (Cleton-Jansen, 2002).

Although there is firm evidence that Wnt-5a has a tumour-suppressive role, a few studies have pointed to Wnt-5a having an oncogenic role in tumours arising from a variety of different tissues (Table 2). Increased expression of Wnt-5a, a hallmark of oncogenesis, was identified in melanoma skin cancer (Da Forno *et al*, 2008), breast cancer cells (Fernandez-Cobo *et al*, 2007), gastric cancer (Kurayoshi *et al*, 2006), pancreatic cancer (Ripka *et al*, 2007), non-small-cell lung cancer (Huang *et al*, 2005) and prostate cancer (Wang *et al*, 2007). Increased expression has been associated with increasing tumour grade (Kurayoshi *et al*, 2006; Da Forno *et al*, 2008), and multivariate analysis showed that expression was an independent risk factor for reduced metastasis-free and overall survival in patients with melanoma (Da Forno *et al*, 2008) or non-small-cell lung cancer (Huang *et al*, 2005). A cancer-promoting function was also shown in UACC 1273 melanoma cancer cells (Weeraratna *et al*, 2002), MKN-74 and MKN-45 gastric cancer cells (Kurayoshi *et al*, 2006) and in PANC1, HT1080, ImimPc1 and MiaPaca pancreatic cancer cell lines (Ripka *et al*, 2007), where overexpression of Wnt-5a promoted cell proliferation and invasion.

The potential role for increased Wnt-5a expression in malignant melanoma has recently been outlined as a study established that nuclear *β*-catenin levels are higher in primary tumours than in metastases and that low expression of nuclear *β*-catenin expression in primary tumours predicts poor survival (Chien *et al*, 2009). This suggests that inhibition of canonical Wnt signalling may be important for progression of malignant melanomas and that the increased expression of Wnt-5a in high-grade tumours may serve to inhibit activation of the canonical signalling pathway and augment cancer growth. Therefore, there is a considerable body of data that supports the hypothesis that Wnt-5a can advance particular cancer types, but is unlikely to be a primary or initiating event.

## Conclusion

Wnt-5a partakes in many of the Wnt-dependent signalling processes used during development, tissue homoeostasis and cancer progression, but its role in the latter is still unclear. Further large-scale studies may help to clarify the role of Wnt-5a, but as it has been shown to elicit different downstream effects depending on receptor availability, these studies are unlikely to achieve clarification if the expression of Wnt-5a is monitored in isolation. We feel that the key will be to identify the most important signalling partners to record. Unfortunately, we do not currently have a comprehensive understanding of how Wnt-5a brings about its effects. Indeed, our understanding of the complexities of Wnt signalling is incomplete (Veeman *et al*, 2003). Recently, a number of studies have used siRNA to identify the genes responsible for controlling canonical Wnt signalling (Major *et al*, 2008), and similar investigations are likely to be completed soon for non-canonical Wnt signalling, specifically the role of Wnt-5a. Once an in-depth understanding of the processes by which Wnt-5a brings about its cellular effects is achieved, it is likely that its role in cancer will be clarified.

In addition to its use as a prognostic indicator, the possibility of Wnt-5a being a target for therapeutics has been raised (Safholm *et al*, 2008). One proposed strategy is the use of peptides to mimic the properties of Wnt-5a (Safholm *et al*, 2008), although in our opinion this approach will require considerable caution because of the wide-ranging roles played by Wnt-5a in the cell. In the cancer cell, for instance, where Wnt-5a has been silenced, introduction of a Wnt-5a mimic may concomitantly activate non-canonical signaling, which also has cancer-promoting properties. Blocking downstream effectors of Wnt-5a may prove more effective, and hence the intense level of research into the therapeutic targeting of Wnt signalling (Ewan and Dale, 2008). With targeting will come a need to screen patients and their tumours for involvement of Wnt-5a and its associated signalling partners before chemotherapy to identify those most likely to benefit. The role of Wnt-5a in cancer and normal tissue is intriguing and the current intense level of research will further our understanding and identify novel targets for therapeutic intervention along with predictive biomarkers for targeted therapy regimes.

## Figures and Tables

**Table 1:** Studies where Wnt-5a has tumour-suppressing effect

**Tissue type (*n*)**	**Detection method**	**Expression in tumour**	**Disease outcome**	**References**
Neuro blastoma (37)	mRNA levels	Reduced expression in some tumours	Reduced expression associated with poor outcome tumours	Blanc *et al* (2005)
Colon cancer primary dukes B (55)	IH	Expression lost or reduced in 50% of tumours	Reduced expression strong predictor of adverse outcome and low expression correlated with shorter survival	Dejmek *et al* (2005a)
Breast cancer (94)	IH	Reduced expression in 56% of breast tumours	Univariate regression analysis showed loss of Wnt-5a expression indicates an increased risk of death	Dejmek *et al* (2005b)
Invasive ductal breast carcinomas (59)	IH	Loss of Wnt-5a expression in 44% tumours	Loss associated with a higher histological grade and loss was an independent predictor of recurrence	Jonsson *et al* (2002)
Thyroid cancer Normal tissue (11) Papillary (12) Anaplastic (5)	IH	Low expression in normal tissue High expression in differentiated tumours but low expression in non differentiated tumours	ND	Kremenevskaja *et al* (2005)
Acute myeloid leukaemias (AML) (10) or ALL (10)	mRNA levels	Wnt-5a absent in 80% of ALL and at low levels or absent in all AML	ND	Liang *et al* (2003)
Hepatocellular carcinoma (92)	IH	Reduction or loss of Wnt-5a protein expression was found in 81% of tumours	Loss was significantly associated with higher tumour stage	Liu *et al* (2008)
Acute lymphoblastic leukaemia (ALL) cell lines (6) patients (307)	Gene methylation	Wnt-5a hypermethylation in all cell lines Hypermethylated in 43% of ALL patients Hypermethylation lead to reduced Wnt-5a mRNA expression	Wnt-5a methylation was an independent prognostic factor predicting disease-free survival	Roman-Gomez *et al* (2007)
Leukaemia Cell lines NL(4), Leukaemia (4) Burket lymphoma (6) peripheral blood mononuclear cells (3) Lymphoblastoid cell lines (3) Burket lymphoma tumours (10) NL tumours (30) Non–Hodgkin's lymphomas(36)	Gene methylation	Wnt-5a highly methylated and mRNA silenced in all cell lines. Methylation of Wnt-5a was shown in 50% of Burket lymphoma, 73% of NL tumours and 31% of Non-Hodgkin's lymphomas but not in normal tissue	ND	Ying *et al* (2007)
Colorectal cancer (29) Normal colon tissues (15)	Gene methylation	Wnt-5a methylation was detected in 48% of CRC tumours, but only in 13% of normal tissue paired normal *P*=0.025)	ND	Ying *et al* (2008)

IH=immunohistochemistry; ND=no data.

**Table 2:** Studies where Wnt-5a has a tumour-promoting effect

**Tissue type (*n*)**	**Detected by**	**Expression in tumour compared with normal**	**Disease outcome**	**References**
Melanoma Primary tumours and matched metastases (59)	IH	Increased expression as disease progressed (*P*=0.013)	Multivariate analysis showed Wnt-5a expression was an independent risk factor for reduced metastasis-free and overall survival	Da Forno *et al* (2008)
Breast cancer cell lines normal tissue (10) Metastatic tissue (9) Normal breast cancer cell lines (11)	mRNA levels	Over expression of Wnt-5A in metastasis-derived breast cancer cells in comparison with normal tissues and to breast cancer cell lines	ND	Fernandez-Cobo *et al* (2007)
Non-small-cell lung cancer (123)	IH	Expression of Wnt-5a in 58% of patients	Wnt-5a expression was associated with reduced overall survival and was a bad prognostic indicator	Huang *et al* (2005)
Gastric cancer (237)	IH	Increased expression detected in 30% of tumours and was frequently seen in tumours of a higher grade	Positivity correlated with advanced stage and poor prognosis	Kurayoshi *et al* (2006)
Pancreatic cancer (16)	IH	Upregulation in 81% of tumours compared with normal tissue	ND —	Ripka *et al* (2007)
Prostate cancer (17)	Gene methylation	Reduced methylation in 65% of tumours	ND —	Wang *et al* (2007)
Melanoma Nevi (8) Primary melanoma (10) Metastases (9)	IH	Low expression 25% of Nevi, expression in 80% of primary melanoma, 89% of metastases showed large regions of expression.	Wnt5a overexpression correlates strongly both to survival and time to the development of metastases	Weeraratna *et al* (2002)

IH=immunohistochemistry; ND=no data.
